# Effects of custom-made 3D-printed insoles on foot and body alignment in stroke survivors: an exploratory pilot randomized controlled trial

**DOI:** 10.7717/peerj.21533

**Published:** 2026-07-13

**Authors:** Juhyun Kim, Ghydaa Anwar, Meeyoung Kim

**Affiliations:** 1 Department of Physical Therapy, College of Health Science, Wonkwang University, Iksan, Jeonbuk, Republic of South Korea; 2 Laboratory of Health Science & Nanophysiotherapy, Department of Physical Therapy, Graduate School, Yongin University, Yongin, Republic of South Korea; 3 Department of Physiotherapy, College of Health Sciences, University of Sharjah, Sharjah, United Arab Emirates; 4 Neuromusculoskeletal Rehabilitation Research Group, Research Institute of Medical and Health Sciences, University of Sharjah, Sharjah, United Arab Emirates

**Keywords:** Stroke, Customized insole, 3D printing, Foot alignment, Postural symmetry, Body alignment, Rehabilitation

## Abstract

**Background:**

Stroke survivors frequently exhibit foot deformities and postural asymmetry, contributing to gait instability and increased fall risk. This exploratory pilot randomized controlled trial examined the effects of custom-made 3D-printed insoles on foot alignment and postural symmetry in ambulatory chronic stroke survivors who did not require ankle-foot orthosis (AFO) support.

**Methods:**

Twenty ambulatory chronic stroke survivors who did not require AFO support were randomly allocated to experimental (*n* = 10) or control (*n* = 10) groups. Prior to enrollment, a physiotherapist assessed each participant’s orthotic needs; those with significant foot drop, ankle instability, or clinical indication for AFO were excluded. Both groups participated in standard rehabilitation (five 30-minute sessions weekly for six weeks). The experimental group additionally wore custom-made 3D-printed insoles during daily activities and therapy sessions. Outcomes included foot alignment (medial longitudinal arch angle, transverse arch angle, arch height index), body alignment (shoulder, pelvic, and knee tilt angles), and dynamic balance via Timed Up and Go test (TUG). Data were analyzed using Wilcoxon signed-rank and Mann–Whitney *U* tests.

**Results:**

The experimental group showed a significant increase in medial longitudinal arch (MLA) angle (mean difference 7.95°, *p* = 0.01) with a significant between-group difference at post-intervention (*p* = 0.03). A between-group difference in shoulder tilt angle was observed at post-intervention (mean difference 1.2°, *p* = 0.03), while no significant within-group change was detected in either group. No significant within-group changes or between-group differences were observed for transverse arch angle, arch height index, pelvic tilt, knee tilt, or TUG performance.

**Conclusions:**

Custom-made 3D-printed insoles improved medial arch height and were associated with a between-group difference in shoulder tilt in selected ambulatory chronic stroke survivors who did not require AFO support. These findings should be interpreted cautiously given the small sample size and exploratory pilot design. Larger definitive trials are needed to confirm these results.

## Introduction

Stroke is a leading cause of long-term disability worldwide, commonly resulting in motor and sensory impairments, muscle weakness, and postural asymmetry that compromise balance and mobility (Hendrickson et al., 2014; Jamal et al., 2018; He et al., 2024). Approximately one-third of stroke patients experience falls during hospitalization, significantly impacting recovery outcomes and quality of life (Teasell et al., 2002; Ullah et al., 2019). Neurological lesions following stroke often alter lower-limb biomechanics, including collapse of the medial longitudinal arch, contributing to foot pain and malalignment (Forghany et al., 2014; Wang et al., 2021).

The foot arch serves as a critical biomechanical foundation, supporting body weight and distributing loads efficiently during gait (Buldt et al. 2018). Arch dysfunction or excessive pronation can impair proprioception, shift the center of mass, and increase postural instability (Cote et al., 2005; Moreno-Barriga et al., 2023). Proper body alignment—defined as the symmetrical arrangement of skeletal segments around the body’s center of gravity—is essential for functional mobility and efficient movement (Shumway-Cook & Woollacott, 2022). Abnormal postural alignment in stroke survivors is associated with increased postural sway and decreased stability (Hendrickson et al., 2014; Zhang & Zheng, 2025).

Non-exercise interventions to improve postural alignment include orthoses, kinesiology taping, and customized insoles (Yang, Heo & Lee, 2015; Kobayashi et al., 2019; Liu et al., 2021). Insoles modify the foot-shoe interface, correct malalignment, and may enhance stability throughout the kinetic chain (Lourenço et al., 2022). Custom-made insoles created from 3D foot scans offer superior fit and mechanical correction compared with prefabricated insoles (Lourenço et al., 2022; Ho et al., 2022). While several studies have evaluated the effects of insoles on gait parameters and plantar pressure distribution in stroke survivors, few have systematically analyzed their impact on both foot and body alignment (Ma et al., 2018; Liu et al., 2021; Fu et al., 2022).

This study investigated whether six weeks of wearing custom-made 3D-printed insoles could improve foot alignment, body alignment, and dynamic balance in ambulatory chronic stroke survivors who did not require AFO support. We hypothesized that improved foot alignment through customized mechanical support would propagate through the kinetic chain, resulting in improved overall postural alignment and functional balance.

## Materials & Methods

### Study design

This was an exploratory pilot randomized controlled trial conducted at M Hospital (Jeonbuk, Republic of Korea) between March and May 2025. The study was designed as a hypothesis-generating pilot investigation to inform the design of a future definitive RCT; accordingly, no a priori power calculation was performed. The study protocol was approved by the Institutional Bioethics Committee of Wonkwang Health Science University (Approval No. ABN01-202412-HR-046) and prospectively registered at ClinicalTrials.gov (NCT06756256).

### Participants

**Figure 1:**
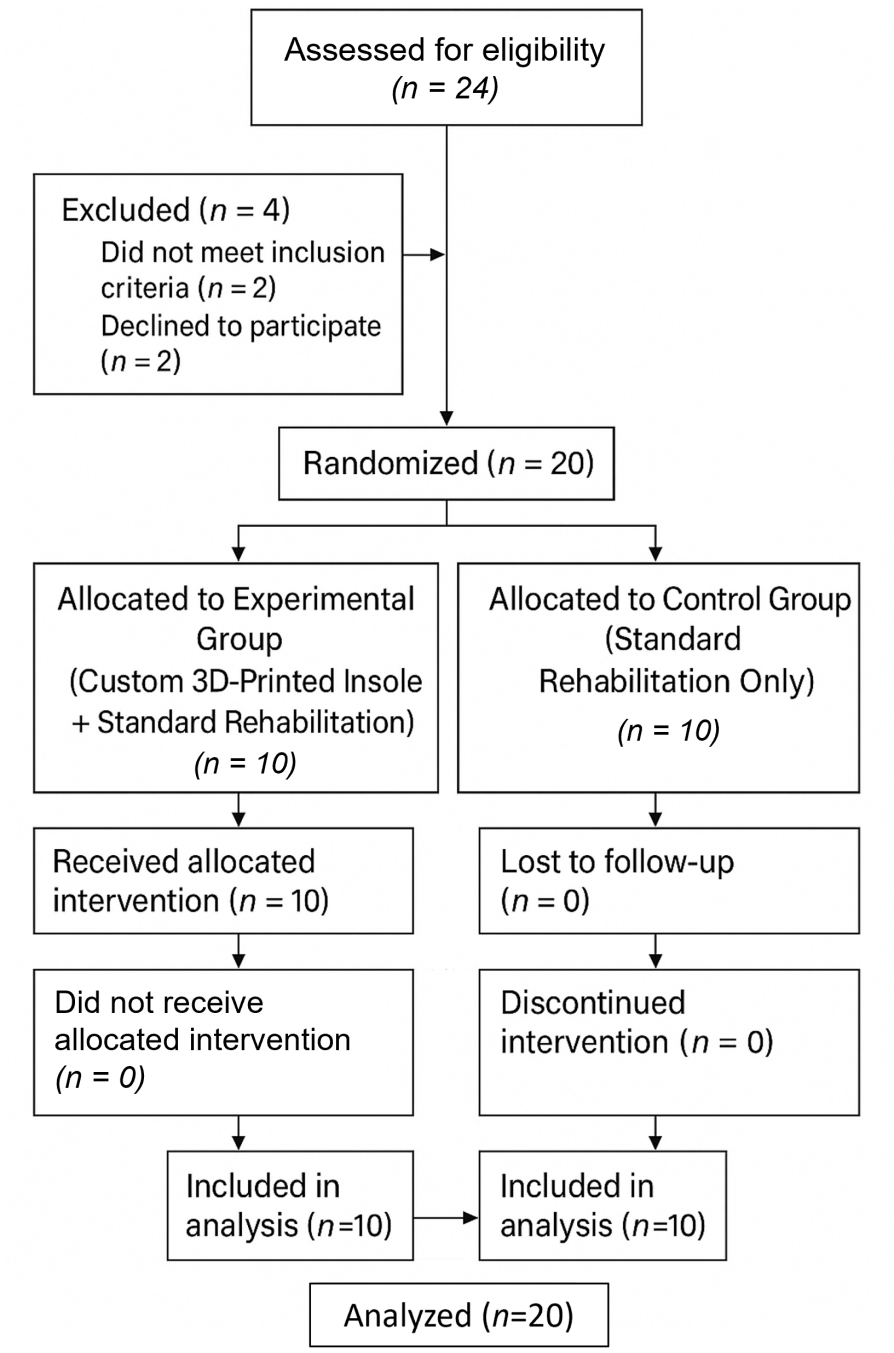
CONSORT flowchart.

Twenty ambulatory chronic stroke survivors hospitalized at M Hospital were recruited and randomly assigned to either an experimental group (*n* = 10) or a control group (*n* = 10) using computer-generated random numbers in sealed envelopes (Fig. 1). The target population was restricted to individuals who were ambulatory and did not require ankle-foot orthosis (AFO)-level structural support, in order to evaluate insoles in their appropriate clinical role as a complementary intervention for optimizing foot-shoe contact and distal alignment. Prior to enrollment, a licensed physiotherapist assessed each participant’s orthotic needs. Participants with notable foot drop, ankle instability, knee-ankle coupling instability, or any other clinical indication for AFO were excluded. AFO use history (current and prior) was recorded for all participants.

### Inclusion criteria

• Diagnosis of stroke more than 1 year prior to enrollment
• Korean Mini-Mental State Examination (MMSE-K) score ≥24
• Ability to ambulate independently with or without assistive devices
• No clinical indication for AFO support as determined by physiotherapist assessment prior to enrollment

Only MMSE-K total scores were used for eligibility screening. The authors have permission to use this instrument from the copyright holders, and no test items or proprietary materials are reproduced in this manuscript (Baek et al., 2016; Song et al., 2020).

### Exclusion criteria

• Other neurological diseases
• Ankle or foot pain limiting mobility
• Severe visual or cognitive impairment
• Medications known to affect balance or vestibular function
• Significant foot drop, marked ankle instability, or knee-ankle instability requiring AFO support
• Current or previous AFO use

All participants provided written informed consent prior to enrollment.

### Intervention

Custom 3D-printed insoles were fabricated using the MediACE Scanner-MS320F foot scanner (Real Dimension Co., South Korea). Each participant’s foot was scanned bilaterally while seated in a standardized position with hips, knees, and ankles positioned at 90 degrees. The scanning system generated corrected 3D models using a proprietary anatomical correction algorithm designed to support the medial longitudinal arch and optimize pressure distribution. Insoles were fabricated using thermoplastic polyurethane (TPU) filament (eTPU-95A, eSUN, China; Shore hardness 95A; diameter 1.75 mm) with a fused filament fabrication (FFF) 3D printer (Style NEO-A31C, Cubicon Co., Ltd., Korea), which provides a build volume of 310 × 310 × 310 mm and supports TPU-based flexible materials. It should be noted that the proprietary nature of the anatomical correction algorithm limits full reproducibility of the insole design. The insoles were intended as a complementary intervention to optimize the foot-shoe interface and assist distal alignment correction, and not as a structural substitute for AFO.

The experimental group wore the custom insoles inside their regular footwear during all daily activities and physiotherapy sessions for six weeks. Participants were instructed to wear the insoles for a minimum of 6 h per day and to record daily wear time in a compliance diary. Both the experimental and control groups participated in a standard rehabilitation program consisting of five 30-minute sessions per week, focusing on gait training, lower-limb strengthening, and foot alignment exercises based on established protocols (Brijwasi & Borkar, 2023).

### Outcome measures

All outcome measures were assessed at baseline and after the six-week intervention period by an assessor blinded to group allocation.

**Foot alignment:** Three parameters were measured using the MediACE Scanner-MS320F: medial longitudinal arch (MLA) angle (degrees), transverse arch angle (TAA) (degrees), and arch height index (AHI) (ratio).

**Body alignment:** Three parameters were measured using the Exbody 770 infrared 3D postural analysis system in a standardized standing position: shoulder tilt angle (STA) (degrees), pelvic tilt angle (PTA) (degrees), and knee tilt angle (KTA) (degrees).

**Dynamic balance:** The Timed Up and Go (TUG) test was performed according to standard procedures (Podsiadlo & Richardson, 1991). Participants were instructed to stand from a seated position, walk three m at a comfortable pace, turn around, walk back, and sit down. Time to complete the task was recorded in seconds.

### Statistical analysis

Statistical analyses were performed using SPSS version 29.0 (IBM Corp., Armonk, NY, USA). Descriptive statistics (mean ± standard deviation) were calculated for all variables. Normality of distribution was assessed using the Shapiro–Wilk test. As data were non-normally distributed, non-parametric tests were employed. Within-group differences between baseline and post-intervention were analyzed using the Wilcoxon signed-rank test. Between-group differences at post-intervention were analyzed using the Mann–Whitney U test. Between-group differences in continuous outcomes are reported as the Hodges–Lehmann median difference estimate, which is the appropriate point estimate for the Mann–Whitney U test. Statistical significance was set at *p* < 0.05 for all analyses.

## Results

### Participant characteristics

Baseline demographic and clinical characteristics did not differ significantly between the experimental and control groups (Table 1), indicating successful randomization. All participants completed the six-week intervention with no dropouts or adverse events. Compliance with insole wear in the experimental group was high, with participants wearing insoles for an average of 7.2 ± 1.1 h per day based on self-reported daily logs.

**Table 1:** Baseline characteristics of participants.

**Characteristic**	**Experimental (*n* = 10)**	**Control (*n* = 10)**	***p*-value**
Age (years)	73.70 ± 10.25	67.10 ± 13.70	0.23
Height (cm)	166.00 ± 10.24	163.00 ± 7.93	0.47
Weight (kg)	58.45 ± 7.83	59.00 ± 8.29	0.88
BMI (kg/m^2^)	21.17 ± 1.74	22.17 ± 2.49	0.31
Sex (male/female)	6/4	5/5	1
Time since stroke (months)	18.60 ± 8.72	24.48 ± 7.84	0.12
Stroke Type (infarct/hemorrhage)	7/3	7/3	1
Affected side (left/right)	6/4	5/5	1
Previous AFO use (yes/no)	0/10	0/10	N/A

**Notes.**

Values are mean ± standard deviation unless otherwise indicated. Independent *t*-tests were used for continuous variables and Fisher’s exact tests were used for categorical variables.

### Foot alignment outcomes

The experimental group showed a significant increase in MLA angle from baseline (154.05 ± 5.66°) to post-intervention (162.00 ± 7.63°) (mean difference 7.95°, *p* = 0.01), indicating improved arch height. The control group showed no significant change in MLA angle (*p* > 0.05). Between-group comparison at post-intervention revealed a significant difference favoring the experimental group (between-group difference 6.10°, *p* = 0.03). No significant within-group or between-group differences were observed for transverse arch angle or arch height index (Table 2).

**Table 2:** Comparison of foot alignment outcomes.

**Variable**	**Group**	**Pre-test** **(mean ± SD)**	**Post-test** **(mean ± SD)**	**Within-group *p*-value**	**Effect size (** ***r*** **)**	**Between-group *p*-value (post)**
MLA (^∘^)	Experimental	154.05 ± 5.66	162.00 ± 7.63	0.01^*^	0.49	0.03^*^
	Control	154.45 ± 7.32	154.33 ± 6.87	0.85	0.05	
TAA (^∘^)	Experimental	120.44 ± 6.30	117.30 ± 6.66	0.17	0.20	0.29
	Control	120.35 ± 6.76	119.99 ± 6.51	0.95	0.05	
AHI	Experimental	0.38 ± 0.03	0.40 ± 0.03	0.86	0.25	0.29
	Control	0.39 ± 0.02	0.39 ± 0.02	0.85	0.05	

**Notes.**

MLA, medial longitudinal arch; TAA, transverse arch angle; AHI, arch height index.

* Significant at *p* < 0.05. Effect sizes (r) are interpreted as small (0.1), medium (0.3), and large (0.5).

### Body alignment outcomes

A between-group difference in shoulder tilt angle was observed at post-intervention (mean difference 1.2°, *p* = 0.03), with the experimental group demonstrating lower asymmetry than the control group. Importantly, no significant within-group change in shoulder tilt was detected in either the experimental or control group; this result therefore reflects a difference between groups at the post-intervention time point and should not be interpreted as insoles having improved shoulder alignment per se. No significant within-group changes or between-group differences were found for pelvic tilt angle or knee tilt angle (Table 3).

**Table 3:** Comparison of body alignment outcomes.

**Variable**	**Group**	**Pre-test** **(mean ± SD)**	**Post-test** **(mean ± SD)**	**Within-group *p*-value**	**Effect size (** ***r*** **)**	**Between-group *p*-value (post)**
STA (^∘^)	Experimental	2.13 ± 1.80	2.14 ± 2.26	0.75	0.49	0.03^*^
	Control	1.20 ± 1.03	0.56 ± 0.72	0.13	0.10	
PTA (^∘^)	Experimental	1.25 ± 1.03	1.43 ± 0.97	0.91	0.10	0.89
	Control	2.30 ± 1.82	1.67 ± 1.50	0.39	0.05	
KTA (^∘^)	Experimental	0.75 ± 0.88	1.43 ± 0.35	0.63	0.05	0.81
	Control	1.40 ± 0.96	1.56 ± 0.72	0.87	0.03	

**Notes.**

STA, shoulder tilt angle; PTA, pelvic tilt angle; KTA, knee tilt angle.

* Significant at *p* < 0.05. Effect sizes (r) are interpreted as small (0.1), medium (0.3), and large (0.5).

### Dynamic balance outcomes

No significant within-group changes or between-group differences were observed in TUG performance after the six-week intervention (Table 4).

**Table 4:** Comparison of dynamic balance (TUG test) outcomes.

**Variable**	**Group**	**Pre-test** **(mean ± SD) seconds**	**Post-test** **(mean ± SD) seconds**	**Within-group *p*-value**	**Effect size (r)**	**Between-group *p*-value (post)**
TUG time	Experimental	29.70 ± 21.23	28.24 ± 20.01	0.68	0.10	0.73
	Control	26.00 ± 7.75	26.40 ± 6.08	0.68	0.05	

**Notes.**

TUG, Timed Up and Go test. Effect sizes (r) are interpreted as small (0.1), medium (0.3), and large (0.5).

## Discussion

This exploratory pilot randomized controlled trial found that custom-made 3D-printed insoles significantly improved medial longitudinal arch angle in ambulatory chronic stroke survivors who did not require AFO support. These findings should be interpreted as hypothesis-generating rather than definitive, given the small sample size and the absence of a priori power calculations. The insoles were positioned as a complementary intervention targeting foot-shoe interface optimization and distal alignment correction in patients who were ambulatory without AFO-level structural support and are not intended as a substitute for AFO management in patients with clinical indications for that intervention. The results are consistent with the concept that distal foot-level corrections may propagate biomechanical effects through the kinetic chain to influence, proximal postural alignment (Sulowska et al., 2019; Shumway-Cook & Woollacott, 2022).

The significant improvement in MLA angle in the experimental group suggests that custom 3D-printed insoles effectively provide mechanical support to the medial arch, correcting the arch collapse commonly observed in stroke survivors. This finding is consistent with previous research demonstrating that foot orthoses can modify foot structure and improve arch support in neurological populations (Pohl & Mehrholz, 2006). The lack of change in transverse arch angle and arch height index may indicate that the insole design primarily targeted medial arch support, or that these parameters require longer intervention periods or more intensive mechanical correction to demonstrate measurable changes.

The between-group difference in shoulder tilt angle at post-intervention requires careful interpretation. This was a between-group difference at a single time point; no significant within-group change in shoulder tilt was observed in either group. The result should therefore not be interpreted as evidence that insoles improved shoulder alignment, but rather as a preliminary signal that warrants further investigation. Furthermore, the clinical relevance of a mean between-group difference of 1.2° in shoulder tilt is uncertain. While the difference was statistically significant (*p* = 0.03), a magnitude of this size may fall below the threshold for clinically meaningful change at the individual patient level. These findings are consistent with kinetic chain theory and provide exploratory evidence that foot-level alignment correction may influence proximal posture (Sulowska et al., 2019; Shumway-Cook & Woollacott, 2022), but replication in a larger, adequately powered trial is necessary before clinical conclusions can be drawn (Pohl & Mehrholz, 2006; Guerra Padilla, Molina Rueda & Alguacil Diego, 2014).

Absence of significant changes in pelvic and knee tilt may reflect: (i) proximal segments requiring targeted trunk/hip stabilization rather than distal-only inputs (Dubey, Karthikbabu & Mohan, 2018) (ii) time-course limits—six weeks may be too brief to shift chronic, compensatory patterns; benefits often consolidate 3 months after training ends (Cabanas-Valdés et al., 2017), and (iii) the need for combined approaches (*e.g.*, pelvic/trunk stabilization + mechanical aids); for example, taping/exercise paradigms improve pelvic tilt and gait in chronic stroke, and duration matters (Jung et al., 2022).

The absence of significant improvement in TUG performance is an important finding that warrants discussion. Several factors may explain this result. First, the six-week intervention duration may have been too short to observe functional balance improvements. Studies reporting significant balance gains in stroke survivors typically employed intervention periods of 8–12 weeks or longer (Vahlberg et al., 2017; Zhou et al., 2024). Second, dynamic balance is a complex construct dependent not only on postural alignment but also on neuromuscular control, sensory integration, muscle strength, and motor planning—factors that insoles alone may not directly enhance (Imbiriba et al., 2020; Kimijanová, Svoboda & Han, 2024). Third, while insoles improved static postural alignment, the transition to dynamic balance improvements during functional tasks may require concurrent neuromuscular training.

Our findings suggest that custom 3D-printed insoles may serve as a practical and accessible adjunct to conventional postural rehabilitation in stroke survivors who do not require AFO-level support. However, insoles should be viewed as one component of a multimodal approach rather than a standalone intervention and the present results must be treated as preliminary pending replication in a larger trial.

### Strengths and limitations

This study has several strengths including the randomized controlled design, objective measurement tools, and high compliance rates. However, several important limitations must be acknowledged. First, this was an exploratory pilot trial with a small sample size (*n* = 20) and no a priori power calculation; the study was therefore likely underpowered to detect small-to-moderate effects, particularly for the TUG test. All findings should be treated as hypothesis-generating. Second, participant characterization was insufficient: data on level of mobility, and use of mobility aids were not systematically collected or reported, limiting comparison with other studies and reducing the interpretability of results. Third, the orthotic eligibility assessment relied on clinical judgment without a standardized, validated AFO screening protocol, which reduces the reproducibility of participant selection. Fourth, the proprietary nature of the anatomical correction algorithm used to design the insoles limits the reproducibility of the intervention. Fifth, some outcome differences, particularly the 1.2° between-group difference in shoulder tilt, may be of limited clinical relevance despite statistical significance. Sixth, the six-week intervention period may have been insufficient to detect changes in all outcomes, particularly dynamic balance. Seventh, we did not include long-term follow-up to assess retention of benefits. Eighth, compliance was monitored through self-report diaries rather than objective means. Finally, the single-center design with a relatively homogeneous patient population limits generalizability to broader stroke populations.

Future research should address these limitations through larger, multicenter trials with longer intervention and follow-up periods. Studies combining 3D-printed insoles with targeted balance training, proprioceptive exercises, or neuromuscular electrical stimulation may reveal synergistic effects on both alignment and functional outcomes (Tariq et al., 2025; Yang et al., 2023). Investigation of dose–response relationships and identification of patient characteristics predicting positive responses to insole interventions would inform clinical decision-making.

## Conclusions

In this exploratory pilot randomized controlled trial, custom-made 3D-printed insoles significantly improved medial longitudinal arch angle in ambulatory chronic stroke survivors who did not require AFO support. A between-group difference in shoulder tilt angle was observed at post-intervention; however, this did not reflect a within-group change in either group and its clinical relevance remains uncertain. No significant effects on dynamic balance were detected over the six-week intervention period. These preliminary findings support further investigation of custom 3D-printed insoles as a complementary adjunct targeting foot-shoe interface optimization and distal alignment correction in selected ambulatory stroke survivors. Given the exploratory pilot design and small sample size, findings should be interpreted cautiously and should not be generalized beyond the study population. A larger, adequately powered definitive RCT is warranted to confirm these results and to clarify the optimal integration of 3D-printed insole technology within stroke rehabilitation programs.

## Supplemental Information

10.7717/peerj.21533/supp-1 Supplemental Information 1 3D scanner dataset

10.7717/peerj.21533/supp-2 Supplemental Information 2 Gait parameter Dataset

10.7717/peerj.21533/supp-3 Supplemental Information 3 CONSORT checklist

## Additional Information and Declarations

### Data Availability

The following information was supplied regarding data availability: The data is available in the Supplemental Files.

### Clinical Trial Registration

The following information was supplied regarding Clinical Trial registration: NCT06756256.

## References

[ref-1] Baek MJ, Kim K, Park YH, Kim SY (2016). The validity and reliability of the Mini-Mental State Examination-2 for detecting mild cognitive impairment and Alzheimer’s disease in a Korean population. PLOS ONE.

[ref-2] Brijwasi T, Borkar P (2023). A comprehensive exercise program improves foot alignment in people with flexible flat foot: a randomised trial. Journal of Physiotherapy.

[ref-3] Buldt AK, Forghany S, Landorf KB, Levinger P, Murley GS, Menz HB (2018). Foot posture is associated with plantar pressure during gait: a comparison of normal, planus and cavus feet. Gait & Posture.

[ref-4] Cabanas-Valdés R, Bagur-Calafat C, Girabent-Farrés M, Caballero-Gómez FM, du Port de Pontcharra Serra H, German-Romero A, Urrútia G (2017). Long-term follow-up of a randomized controlled trial on additional core stability exercises training for improving dynamic sitting balance and trunk control in stroke patients. Clinical Rehabilitation.

[ref-5] Cote KP, Brunet ME, Gansneder BM, Shultz SJ (2005). Effects of pronated and supinated foot postures on static and dynamic postural stability. Journal of Athletic Training.

[ref-6] Dubey L, Karthikbabu S, Mohan D (2018). Effects of pelvic stability training on movement control, hip muscle strength, walking speed and daily activities after stroke: a randomized controlled trial. Annals of Neurosciences.

[ref-7] Forghany S, Nester CJ, Tyson SF, Preece S, Jones RK (2014). The effect of stroke on foot kinematics and the functional consequences. Gait & Posture.

[ref-8] Fu JC, Chen YJ, Li CF, Hsiao YH, Chen CH (2022). The effect of three-dimensional printing hinged ankle-foot orthosis for equinovarus control in stroke patients. Clinical Biomechanics.

[ref-9] Guerra Padilla M, Molina Rueda F, Alguacil Diego IM (2014). Effect of ankle-foot orthosis on postural control after stroke: a systematic review. Neurología.

[ref-10] He Q, Wang W, Zhang Y, Xiong Y, Tao C, Ma L, Ma J, You C, Wang C (2024). Global, regional, and national burden of stroke, 1990–2021: a systematic analysis for Global Burden of Disease 2021. Stroke.

[ref-11] Hendrickson J, Patterson KK, Inness EL, McIlroy WE, Mansfield A (2014). Relationship between asymmetry of quiet standing balance control and walking post-stroke. Gait & Posture.

[ref-12] Ho M, Nguyen J, Heales L, Stanton R, Kong PW, Kean C (2022). The biomechanical effects of 3D printed and traditionally made foot orthoses in individuals with unilateral plantar fasciopathy and flat feet. Gait & Posture.

[ref-13] Imbiriba LA, Correia MRA, Farias SG, Silva JM, da Nobrega Ferreira I, Garcia MAC, Sperandei S, Macedo ARD (2020). What we know so far about postural balance training: an exploratory scoping review of nomenclature and related issues. Journal of Bodywork and Movement Therapies.

[ref-14] Jamal K, Leplaideur S, Rousseau C, Chochina L, Moulinet-Raillon A, Bonan I (2018). Disturbances of spatial reference frame and postural asymmetry after a chronic stroke. Experimental Brain Research.

[ref-15] Jung KS, Jung JH, In TS, Cho HY (2022). Effects of pelvic stabilization training with lateral and posterior tilt taping on pelvic inclination, muscle strength, and gait function in patients with stroke: a randomized controlled study. BioMed Research International.

[ref-16] Kimijanová J, Svoboda Z, Han J (2024). Editorial: sensory control of posture and gait—integration and mechanisms to maintain balance during different sensory conditions. Frontiers in Human Neuroscience.

[ref-17] Kobayashi T, Orendurff MS, Hunt G, Gao F, LeCursi N, Lincoln LS, Foreman KB (2019). The effects of alignment of an articulated ankle-foot orthosis on lower limb joint kinematics and kinetics during gait in individuals post-stroke. Journal of Biomechanics.

[ref-18] Liu YT, Tsai HT, Hsu CY, Lin YN (2021). Effects of orthopedic insoles on postural balance in patients with chronic stroke: a randomized crossover study. Gait Posture.

[ref-19] Lourenço BM, Magalhães FA, Vieira FM, Reis CK, Costa HS, Araújo VL, Richards J, Trede R (2022). An exploration of the effects of prefabricated and customized insoles on lower limb kinetics and kinematics during walking, stepping up and down tasks: a time series analysis. Gait & Posture.

[ref-20] Ma CC, Rao N, Muthukrishnan S, Aruin AS (2018). A textured insole improves gait symmetry in individuals with stroke. Disability and Rehabilitation.

[ref-21] Moreno-Barriga OS, Romero-Morales C, Becerro-de Bengoa-Vallejo R, Losa-Iglesias ME, Gómez-Salgado J, Caballero-López J, Vidal-Valverde LC, López-López D (2023). Effects of foot structure type on core stability in university athletes. Life.

[ref-22] Podsiadlo D, Richardson S (1991). The timed “Up & Go”: a test of basic functional mobility for frail elderly persons. Journal of the American Geriatrics Society.

[ref-23] Pohl M, Mehrholz J (2006). Immediate effects of an individually designed functional ankle-foot orthosis on stance and gait in hemiparetic patients. Clinical Rehabilitation.

[ref-24] Shumway-Cook A, Woollacott MH (2022). Motor control: translating research into clinical practice. 6th edition.

[ref-25] Song M, Jahng S, Kim SY, Kang Y (2020). A normative study of the full version of story memory in the Korean-Mini Mental State Examination, 2nd edition: expanded version (K-MMSE-2: EV). Dementia and Neurocognitive Disorders.

[ref-26] Sulowska I, Mika A, Ł Oleksy, Stolarczyk A (2019). The influence of plantar short foot muscle exercises on the lower extremity muscle strength and power in proximal segments of the kinematic chain in long-distance runners. BioMed Research International.

[ref-27] Tariq S, Waris A, Gilani SO, Mushtaq S, Awais Q, Iqbal J, Mushtaq K, Khan NB (2025). Evaluation of balance and orthotic gait training techniques for rehabilitation in hemiplegic stroke patients. Scientific Reports.

[ref-28] Teasell R, McRae M, Foley N, Bhardwaj A (2002). The incidence and consequences of falls in stroke patients during inpatient rehabilitation: factors associated with high risk. Archives of Physical Medicine and Rehabilitation.

[ref-29] Ullah S, Al-Atwi M, Qureshi AZ, Tantawy SS, Ilyas A, Wunderlich CA (2019). Falls in individuals with stroke during inpatient rehabilitation. Neurosciences.

[ref-30] Vahlberg B, Cederholm T, Lindmark B, Zetterberg L, Hellström K (2017). Short-term and long-term effects of a progressive resistance and balance exercise program in individuals with chronic stroke: a randomized controlled trial. Disability and Rehabilitation.

[ref-31] Wang J, Qiao L, Yu L, Wang Y, Taiar R, Zhang Y, Fu W (2021). Effect of customized insoles on gait in post-stroke hemiparetic individuals: a randomized controlled trial. Biology.

[ref-32] Yang SR, Heo SY, Lee HJ (2015). Immediate effects of kinesio taping on fixed postural alignment and foot balance in stroke patients. Journal of Physical Therapy Science.

[ref-33] Yang Q, Song Y, Shangguan Y, Li J (2023). Research on proprioceptive-electrical stimulation insole based on flexible sensor array.

[ref-34] Zhang T, Zheng J (2025). Enhancing postural control in stroke patients: advances in mechanisms and functional recovery analysis of core stability training. Neurological Science.

[ref-35] Zhou Y, Ren H, Hou X, Dong X, Zhang S, Lv Y, Li C, Yu L (2024). The effect of exercise on balance function in stroke patients: a systematic review and meta-analysis of randomized controlled trials. Journal of Neurology.

